# Basic and Translational Models of Cooperative Oncogenesis

**DOI:** 10.3390/ijms21165919

**Published:** 2020-08-18

**Authors:** Helena E. Richardson, Julia B. Cordero, Daniela Grifoni

**Affiliations:** 1 Department of Biochemistry and Genetics, La Trobe Institute for Molecular Science, La Trobe University, Melbourne, VIC 3086, Australia; 2 Wolfson Wohl Cancer Research Centre, Institute of Cancer Science, University of Glasgow, Glasgow G61 1QH, UK; julia.cordero@glasgow.ac.uk; 3 Department of Life, Health and Environmental Sciences, University of L’Aquila, I-67100 L’Aquila, Italy; daniela.grifoni@univaq.it

Cancer is a complex set of diseases involving genetic or epigenetic changes within cells, as well as interactions between the developing tumour cells and their microenvironment, which leads to uncontrolled tumour growth, altered differentiation, local invasion and metastasis to distant sites [1,2]. Molecular changes in oncogenes or tumour suppressor genes promote various cancer hallmarks, including the continued proliferation, inhibition of differentiation, inhibition of apoptosis, changes to metabolism, evasion of the immune system and the promotion of invasion/metastasis [3,4]. Although many oncogenes and tumour suppressor gene mutations promote more than one hallmark of cancer, several mutations are required to generate malignant cancers, a process referred to as cooperative oncogenesis/tumourigenesis [5,6]. Through decades of research, we have gained much knowledge on key molecular events and processes involved in the formation of cancer, and much of this knowledge has stemmed from investigations using model organisms, such as the mouse and the vinegar fly, *Drosophila melanogaster*, in addition to in vitro cell line studies. In this Special Issue, we present a collection of original research papers on various aspects of cancer research utilising human cell lines [7,8], or in vivo using *Drosophila* as a model system [9,10], as well as reviews highlighting the *Drosophila* model organism in cancer research [11,12,13,14,15]. *Drosophila* is a particularly useful model organism for the study of cancer mechanisms, because it has a rapid life cycle and is genetically manipulatable and since cancer genes and signalling pathways are highly conserved between humans and *Drosophila*, and interactions between tumour cells and surrounding normal cells can be readily examined in *Drosophila* tissues [6,16,17,18,19,20].

In a research paper pertaining to in vitro models of cancer, Di Giorgio et al. [8] analyse the transcriptional response to the expression of three key oncogenes (*RAS*, *MYC*, and *HDAC4*) in human fibroblasts, revealing common signalling pathways that are deregulated by these genes, and suggesting potential therapeutic avenues for the treatment of cancers driven by these oncogenes. In the second research paper on this topic, Mayer et al. [7] focus on human pancreatic cancer, where they observe in tissue sections the infiltration of Th17-like T cells expressing IL21 and IL26, and the expression of receptors for IL21 and IL26 in the pancreatic epithelial cells. They show in human pancreatic cell lines that IL21 and IL26 signal through ERK1/2 and STAT, which leads to increased tumour cell growth in colony forming assays.

In a research paper utilising the *Drosophila* model, Parniewska and Stocker [9] identify the novel splicing factor, SF2, which is essential for the survival and hyperproliferation of tissues that upregulate the target of rapamycin complex 1 (TORC1), a protein kinase involved in cellular growth that is upregulated in many cancers [21,22]. The identification of SF2 as a key conserved target of TORC1, which is required for early tumour growth in *Drosophila*, provides a potential new approach to develop anti-cancer therapies for tumours with upregulated TORC1 activity, such as those carrying loss of function of *PTEN* (phosphatase and tensin homolog deleted on chromosome ten) or constitutively active mutations in *PI3K* (phospho inositol 3 kinase) [23,24,25]. In a second research paper utilising the *Drosophila* model, Hamaratoglu and Atkins [10] undertook an analysis of published transcriptional data from various *Drosophila* imaginal disc epithelial cell models of cancer, and found that the JNK stress response pathway [26,27], and JAK/STAT [28,29], Hippo [30,31] and Notch [32,33] tissue growth signalling pathways are commonly deregulated. This important meta-analysis has opened-up new potential avenues of research to examine the cooperative interactions between these signalling pathways using model organisms, as well as to assess the co-dependency of these conserved pathways in human cancers.

The reviews in this Special Issue highlight the power of using *Drosophila* as an in vivo model system to study various aspects of cancer research, from its application in the study of the function of specific genes/pathways in cancer [11,15], to understanding particular cellular processes in cancer [13,14], and for functional analyses of cancer Omics data [12]. Sechi et al. [15] review the mechanisms of the Golgi phosphoprotein 3 (*GOLPH3*) oncogene in cancer, covering research on human cancer samples, in vitro cell line analyses and *Drosophila* in vivo analyses. Carmena [11] reviews the involvement of the Scribble cell polarity module [34] in asymmetric cell division (ACD) of *Drosophila* neural stem cells in tumourigenesis, highlighting the importance of correct ACD for appropriate differentiation and exit from the cell cycle. Casas-Tintó and Portela [14] review the involvement of specialised cellular extensions, termed cytonemes, in cell–cell communication and in tumourigenesis. Cytonemes have recently been shown to be highly important in the development of glioblastoma and the associated neural degeneration that occurs in *Drosophila* models and also in the human disease [35], and there is accumulating evidence for their role in tumourigenesis in other cancer types. Newman and Gregory [13] review the connection between chromosomal aberrations (aneuploidy) and metabolic changes, highlighting the role of oxidative stress and particularly reactive oxygen species (ROS) in this process. Finally, Bangi [12] reviews how *Drosophila* can be utilised to functionally analyse the vast amount of human cancer Omics data that is currently being generated, in order to validate key genes/pathways that contribute to cancer, to build new models to interrogate cancer mechanisms, and to screen for novel cancer therapeutics. *Drosophila* has already proven its worth in identifying novel drugs that target particular types of human cancers, such as multiple endocrine neoplasia type 2 (MEN2), colorectal and non-small cell lung cancers [36,37,38,39,40,41,42], and undoubtably, the development of more sophisticated *Drosophila* models that incorporate additional genetic lesions will enable better modelling of human cancers and new anti-cancer drug discovery.

This first iteration of this Special Issue on basic and translational models of cooperative oncogenesis presents only a snapshot of the vast amount of research into cancer currently being conducted worldwide, yet it highlights the important contribution of the simple multicellular model organism, *Drosophila*, to our current understanding of cancer. Undoubtably, further primary research papers and literature reviews for future iterations of this Special Issue will highlight new cancer genes/pathways and processes involved in cancer, additional in vivo models of cancer (such as worms, zebra fish, and mice), and novel approaches for the understanding of cancer mechanisms and for developing new cancer therapies.
